# Multi-compositional MRI evaluation of repair cartilage in knee osteoarthritis with treatment of allogeneic human adipose-derived mesenchymal progenitor cells

**DOI:** 10.1186/s13287-019-1406-7

**Published:** 2019-10-21

**Authors:** Xinxin Zhao, Jingjing Ruan, Hui Tang, Jia Li, Yingxuan Shi, Meng Li, Suke Li, Cuili Xu, Qing Lu, Chengxiang Dai

**Affiliations:** 1 Department of Radiology, Ren Ji Hospital, School of Medicine, Shanghai Jiao Tong University, No. 160, Pujian Road, Shanghai, 200127 China; 2 Department of Rheumatology, Ren Ji Hospital, School of Medicine, Shanghai Jiao Tong University, No. 160, Pujian Road, Shanghai, 200127 China; 3 Cellular Biomedicine Group, Inc., No. 85 Faladi Road, Building 3, Zhangjiang, Pudong New Area, Shanghai, 201210 China

**Keywords:** Multi-compositional MRI sequences, Knee osteoarthritis, Composition alternations, Repair cartilage, Human adipose-derived mesenchymal progenitor cells

## Abstract

**Background:**

We used multimodal compositional magnetic resonance imaging (MRI) techniques, combined with clinical outcomes, to differentiate the alternations of composition in repair cartilage with allogeneic human adipose-derived mesenchymal progenitor cells (haMPCs) in knee osteoarthritis (KOA) patients.

**Methods:**

Eighteen patients participated a phase I/IIa clinical trial. All patients were divided randomly into three groups with intra-articular injections of haMPCs: the low-dose (1.0 × 10^7^ cells), mid-dose (2.0 × 10^7^), and high-dose (5.0 × 10^7^) groups with six patients each. Compositional MRI examinations and clinical evaluations were performed at different time points.

**Results:**

Significant differences were observed in quantitative T1rho, T2, T2star, R2star, and ADC measurements in patients of three dose groups, suggesting a possible compositional changes of cartilage with the treatment of allogeneic haMPCs. Also significant reduction in WOMAC and SF-36 scores showed the symptoms might be alleviated to some extent with this new treatment. As regards sensibilities of multi-parametric mappings to detect compositional or structural changes of cartilage, T1rho mapping was most sensitive to differentiate difference between three dose groups.

**Conclusions:**

These results showed that multi-compositional MRI sequences might be an effective tool to evaluate the promotion of the repair of cartilage with allogeneic haMPCs by providing information of compositional alterations of cartilage.

**Trial registration:**

Clinicaltrials, NCT02641860. Registered 3 December 2015.

## Introduction

Knee osteoarthritis (KOA) is characterized mainly by arthralgia, stiffness, loss of mobility with occasional effusion, and variable degrees of local inflammation [1, 2]. As one of the most prevalent chronic degenerative diseases, KOA is associated with the progressive degradation of articular cartilage, meniscus, synovium, ligaments, bone, muscles, and tendons [3, 4]. The available therapies of KOA are pharmacologic [5], non-pharmacologic [6], and even knee replacement for severe osteoarthritis [7]. Unfortunately, these traditional treatments demonstrate only relieve symptoms [8, 9], whereas they cannot reverse the pathological process or regenerate articular cartilage [10].

Stem cells have been used to regenerate cartilage defects for decades [11, 12]. Recently, growing evidence suggests therapeutic potential of mesenchymal stem/progenitor cells (MSC/MPC) for cartilage repair and regeneration by their ability to differentiate into chondral tissue [9, 11–14], for instance encouraging results of treatments with intra-articular injection of adipose-derived mesenchymal progenitor cells (AD-MPCs) in preclinical or clinical studies [15–17], and autologous bone marrow-derived mesenchymal stem cells (BM-MSCs) [18–20] or adipose tissue-derived MSCs (AD-MSCs) [21, 22] in clinical trial. The efficacy of intra-articular injection of allogeneic human adipose-derived MPCs (haMPCs) in the knee has been verified in OA animal models [15], yet not in patients. Nonetheless, most of these studies focus on traditional methods, including clinical, radiography, arthroscopy, and histological assessment [2, 15, 18], which are insensitive to ultra-structural tissue alterations, and can only detect gross morphological changes occurred over long periods [10, 23].

Magnetic resonance imaging (MRI), with its excellent soft tissue contrast and ability to acquire morphological and biochemical data [3, 10, 23, 24], provides a novel non-invasive technology to directly visualize joint tissues associated with OA [3, 24]. Several MRI techniques, known as “compositional imaging” of cartilage, that were used to selectively demonstrate the glycosaminoglycan (GAG) and collagen fiber network of the extracellular matrix (ECM) include T2 mapping, T1rho mapping, T2star mapping, and diffusion weighted imaging (DWI). T2 mapping is performed with a multi-echo multi-spin sequence to measure T2 relaxation times of different tissues which is influenced by the orientation of the collagen framework and the restricted water mobility [25]. Similar to T2 relaxation, T1rho is spin-lattice relaxation with an additional RF pulse after the magnetization is tipped into a transverse plane. In addition to GAG and collagen-specific changes, T1rho may reflect nonspecific changes in the cartilage ECM [26, 27]. T2star relaxation time (the reciprocal of R2 star) is acquired with the de-phasing effect of gradient echo, which depends on local field inhomogeneities and susceptibility caused by tissue changes in articular cartilage [28]. DWI provides valuable information regarding the structure and organization of cartilage by applying multiple diffusion-sensitive gradients to evaluate water molecule orientation. Apparent diffusion coefficient (ADC) reflects the motion of water molecules within the cartilage ECM, with increased diffusivity linked to structural degradation of the ECM [29].

Recently, Guermazi et al. have reported these compositional MRI techniques play an increasingly important role for the evaluation of cartilage degeneration in osteoarthritis and detection of the compositional and structural difference of the cartilage after treatments [3]. Aurelio et al. have used T2 mapping to evaluate articular cartilage quality on patients with KOA with allogeneic MSC treatment [18]. Chris et al. have evaluated the size, depth of cartilage defect, and signal intensity of regenerated cartilage with high-resolution MR imaging for patients with treatment of intra-articular injection of MSCs [2]. And Felix et al. have predicted knee replacement with quantitative MRI measures of cartilage [7]. However, most of these studies attempted to detect complicated changes in composition and structure of cartilage only by single or two MRI measurements that were specific to changes in the cartilage. Given the limitations in specificities of different MRI parameters, multimodal MRI methods have been used to predict compositional structure of the cartilage. Multi-parametric data using different MRI sequences can provide detailed information about the underlying processes of cartilage repair and can provide additional specificity.

To differentiate the alternations of cartilage composition, we conducted a MRI study with multiple compositional sequences in a proof-of-concept phase I/IIa clinical trial to assess the efficacy of intra-articular injection of allogeneic haMPCs in patients with KOA. The purpose of this study was to determine the ability of multi-compositional MRI techniques to evaluate the potential of repair cartilage with allogeneic haMPCs in patients with KOA.

## Materials and methods

### Patients

This was a randomized and double-blind phase I/IIa clinical trial, which was conducted between March 2016 and August 2017 at Ren Ji Hospital, School of Medicine, Shanghai Jiao Tong University, in China and registered at ClinicalTrials.gov (NCT02641860). This study received approval from the local ethics committee, and all participants signed a consent form. Twenty-two patients with KOA were recruited, and all subjects were informed of the protocol design before providing written informed consent. Detailed inclusion and exclusion criteria are reported in Table 1. All patients received an intra-articular injection of allogeneic haMPCs at the base time and were followed up at 48 weeks. Eighteen patients were subjected for statistical analysis. Four patients were not included due to loss of follow-up. Demographic and clinical information on subjects is given in Table 2. Disease severity and movement symptom severity of OA were assessed using the Western Ontario and McMaster Universities Osteoarthritis Index (WOMAC) pain scale [24] as graded by a movement disorder specialist and the Medical Outcomes Short-Form-36 questionnaire (SF-36) [30].

**Table 1:** Inclusion and exclusion criteria

Inclusion criteria	
1. Grade II–III osteoarthritis, identified by two different observers, according to the Kellgren-Lawrence grading scale	
2. Hematological and biochemical analyses with no significant alterations that contraindicate intervention	
3. Aged 18 to 70 years old, males or females	
4. Diagnostic course of knee osteoarthritis for more than 6 months and less than 10 years	
5. An average pain intensity of grade 3 or more (less than 8) on a 10-point visual analog scale (VAS)	
6. Informed written consent provided by the patient	
Exclusion criteria	
1. History of one or more drug allergies and two or more food allergies	
2. Obesity with a body mass index > 30 (calculated as mass in kg/height in m^2^)	
3. Neoplasia	
4. Signs of infection or positive serology for HIV, hepatitis, or syphilis	
5. Congenital or acquired diseases leading to significant knee deformities that may interfere with cell application or the interpretation of results	
6. Pregnancy or breastfeeding	
7. Immunosuppression	
8. Intra-articular injection of any drug during the previous 3 months	
9. Participation in another clinical trial or treatment with another investigational product within 30 days prior to inclusion in the study	
10. Other conditions that may, according to medical criteria, discourage participation in the study

**Table 2:** Demographic information on subjects

	Low dose (*n* = 6)	Mid dose (*n* = 6)	High dose (*n* = 6)	*F*	*P* value
Age, years	52.05 ± 11.64	59.58 ± 10.24	52.69 ± 8.72	0.95	0.41
Gender, female (male)	4 (2)	5 (1)	4 (2)	0.48	0.79
Height, cm	165.50 ± 6.44	159.83 ± 6.77	163.60 ± 4.10	1.86	0.19
Weight, kg	71.17 ± 8.84	60.83 ± 10.23	64.71 ± 5.09	2.25	0.14
BMI, kg/m^2^	25.63 ± 1.93	23.73 ± 2.94	24.08 ± 1.44	1.21	0.33
KLG, at base time					
0–1	0	0	0		
2	3	3	2		
3	3	3	3		
4	0	0	0		

### haMPC preparation

#### Cell isolation

The isolation and characterization of haMPCs followed the conventional methods described in a previous report [15]. All cell product manufacturing procedures were performed under Good Manufacturing Practice conditions. Strict quality control and quality assurance measures were applied. Human adipose-derived MSCs were obtained from three healthy donors that were subjected to allogeneic MSC transplantation and produced more cells than needed for clinical trial. The characterization of the adipose-derived mesenchymal stem cells from three donors is concluded in Table 3. Subcutaneous adipose tissues were harvested from abdominal subcutaneous fat by liposuction with local anesthetic. The lipoaspirate (50 g), obtained from each volunteer, was washed three times with phosphate-buffered saline (PBS) to remove the blood cells and tissue debris. Subcutaneous adipose tissues were digested with Dulbecco’s modified Eagle’s medium (DMEM; Invitro-gen) containing 0.1% w/v collagenase A type I (Sigma, St. Louis, MO, USA) under gentle agitation for 60 min at 37 °C. The digested tissues were centrifuged at 1000 rpm for 8 min to obtain a pellet and were filtered through a 100-μm nylon filter (BD Biosciences, Mississauga, ON, Canada) to remove cellular debris.

**Table 3:** The characterization of the adipose-derived mesenchymal stem cells from three donors

Description	Characterization		
Donor			
No.	1#	2#	3#
Sex	Male	Female	Female
Age (yes)	32	23	26
Volume of adipose (ml)	150	100	100
Cell			
Morphology	Cells are adherence to plastic and in spindle shape with large oval nuclei		
Cell marker	Positive marker (CD90, CD73, CD105) > 95% +; negative marker (HLA-DR, CD14, CD45) < 2%		
Potency of chondrogenic differentiation		Positive	
Viability		> 80%	
Endotoxin		< 4 EU/ml	
Sterility		Negative	
Mycoplasma		Negative	

#### Cell culture and expansion

The isolated adipose stem cells were seeded on T-75 culture flasks (inoculation density: 50 ml liposuction fat isolated cells were inoculated into four T-75 culture flasks) and maintained at 37 °C/5% CO2 in serum-free medium for 5~7 days until confluent (passage 0). After digestion with trypsin (0.125%) combined with EDTA (0.01%) for 1.5~2.5 min, cells were passaged at 5 × 10^3^/cm^2^ in the same serum-free proliferation media up to passage 4 for clinical trial.

#### Cell cryopreservation

When the cells reached 85~ 90% confluency, they were digested by the same method mentioned above and washed with PBS to remove nonadherent cells. The haMPCs were finally suspended in a serum-free cryopreservation solution at a cell concentration of 0.5~2 × 10^7^ cells/ml and immediately cryopreserved in liquid nitrogen. And then the cryopreserved cells were thawed before transplantation. The trypan blue viability of cryopreserved and thawed cells reached 80% and 70% respectively. In our clinical trial, participants used cell products twice at day 0 and day 21, each time after resuscitation of passage 4 cryopreserved cells.

#### Suspension transportation and storage

The thawed cell suspension was transported to the clinical research center at 2~6 °C and transferred to a refrigerator at 2–8 °C for storage. The cell suspension will be transplanted to the participants within 24 h.

### Flow cytometry and trilineage differentiation in vitro

Selection of haMPCs was based on their capacity to adhere to the surface of plastic culture flasks. A 5-color flow cytometric analysis was performed using an EPICS XL flow cytometer (Beckman Coulter, Palo Alto, CA, USA). Detailed methods of induction to differentiation were as our previous work [15, 31]. Using flow cytometric analysis and differentiation, the passage 4 of haMPCs was confirmed as stem cells mainly by a positive expression of CD29, CD73, CD90, and CD49d and negative expression of CD14, CD34, CD45, Actin, and HLA-DR. Cell viability and survival were determined by trypan blue staining and exclusion test, and the rate was set at > 80%. In addition, the haMPCs could differentiate to fat, bone, and cartilage tissue cells and inhibit the proliferation of T cells that have the same characteristics as MSCs. Detailed information has been reported in the supplementary appendix in our previous study [15, 31].

### Preclinical toxicity and chronic tumorigenicity in vivo

We conducted preclinical studies after participant enrollment and liposuction. The preclinical toxicity and chronic tumorigenicity of haMPCs in vivo followed the conventional methods described in previous reports [15, 31]. We observed none of mice severely ill or died at any time before the experimental end point. There was no abnormal reaction during the study period [15, 31].

### Quality control

To avoid issues related to bovine serum protein, such as prion-related encephalopathy or other xenogeneic infections, we used a potent serum-free medium (Cellular Biomedicine Group, Inc., Shanghai, China) for culture and expansion of the stem cells [32]. To assess safety, we examined the quality control of the final haMPC preparation and its adverse effects after administration. For quality control, the haMPC preparations were checked for cell survival by trypan blue staining method, genetic level by short tandem repeat (STR) [33], identification of cell surface markers by method of fluorescence-activated cell sorting (FACS), sterility test by direct inoculation according to the 2015 version of the Chinese Pharmacopoeia (Ch.P.2015), and endotoxin contamination by gel method according to the Ch.P.2015.

### Study design

**Fig. 1:**
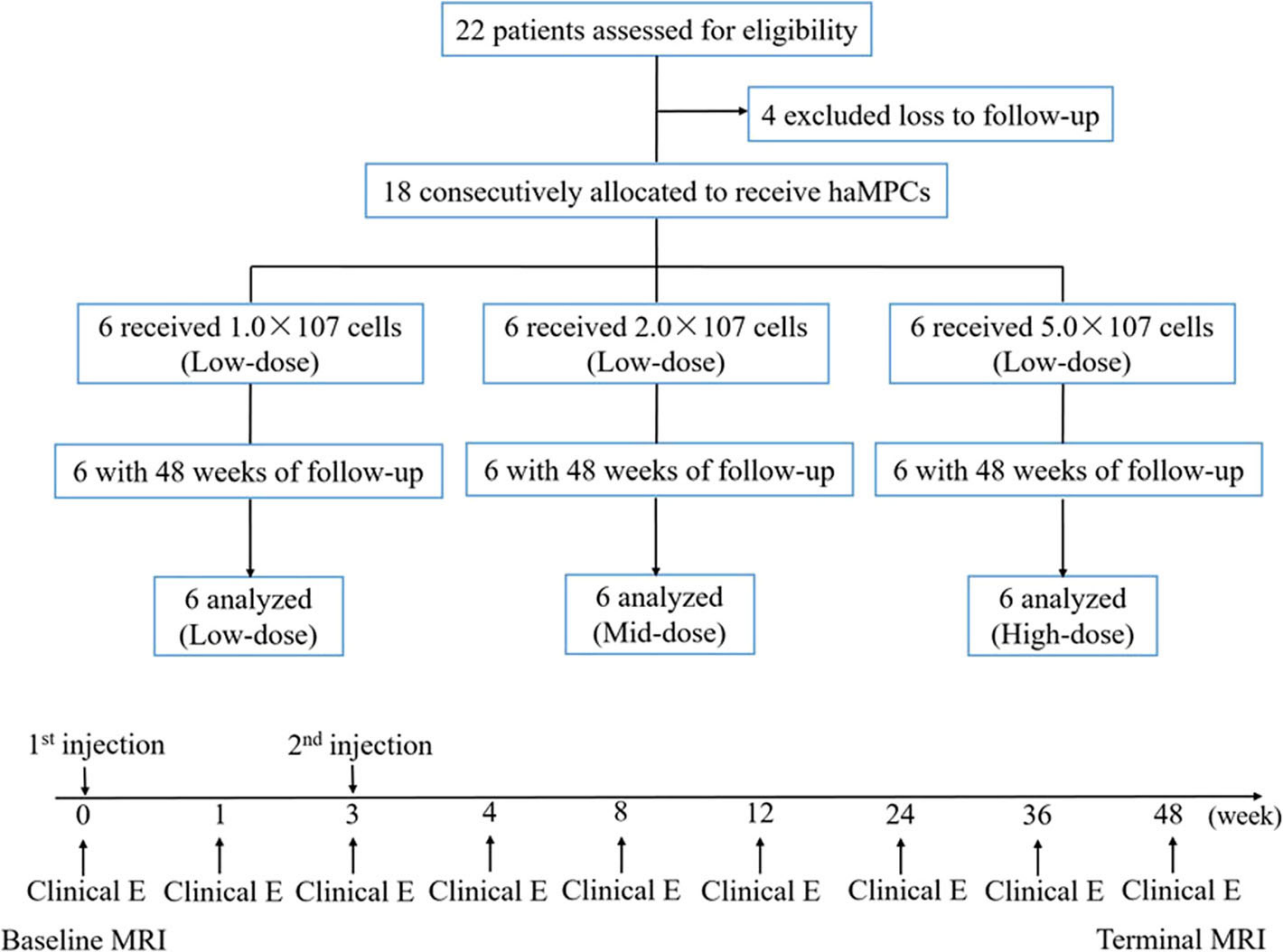
Study flow diagram. *Abbreviations*: haMPCs, human adipose-derived mesenchymal progenitor cells; Clinical E, clinical evaluation; MRI, magnetic resonance imaging

Eighteen patients were divided randomly into three groups according to the subsequent intra-articular injections which they received: the low-dose (1.0 × 10^7^ cells), mid-dose (2.0 × 10^7^), and high-dose (5.0 × 10^7^) groups with six patients each. The overview of the total experimental design is shown in Fig. 1. Compositional MRI examinations were performed at 1 day before the first injection to collect the base time point (baseline) and at 48 weeks to collect terminal point. In addition to the baseline and terminal points, clinical evaluations including VAS, WOMAC, and SF-36 questionnaire were also conducted at 1, 3, 4, 8, 12, 24, 36, and 48 weeks after the first injection.

### MR methods

All subjects were studied on a clinical 3T MR imaging system (Signa HDx; GE Healthcare, Milwaukee, WI, USA) with a dedicated 8-channel knee coil. MRI examination consists of conventional and quantitative scanning. Sagittal T1-weighted (TR/TE 560 ms/10.2 ms), two-dimensional proton density–weighted (2260 ms/30 ms), and fat-saturated fast spin-echo T2-weighted (2600 ms/68 ms) in the three orthogonal planes were acquired for screening other joint diseases. Quantitative MR images included three-dimensional spoiled gradient recalled echo (3D SPGR) with fat suppression, T1rho mapping, T2 mapping, T2star mapping, and diffusion weighted imaging (DWI). In the T1rho mapping sequence, the amplitude of the spin-lock pulse was 500 Hz, spin lock durations = 10/20/30/50 ms. The T2 maps were obtained from the multi-echo spin-echo sequence with the following parameters: TR = 1125 ms, TE1 = 7.6 ms, ΔTE = 6 ms, echo number = 8, FOV =160 × 160 mm^2^, matrix resolution = 320 × 192, slice thickness = 4 mm, and number of slices = 16. The T2star (or R2star) maps were obtained from the multi-echo gradient echo sequence with the following parameters: TR = 60 ms, TE1 = 6.8 ms, ΔTE = 6.8 ms, echo number = 8, flip angle = 15°, FOV = 180 × 180 mm^2^, matrix resolution = 384 × 320, slice thickness = 1.2 mm, and number of slices = 128. The DWI data were acquired from single-shot spin-echo echo planar imaging (ss-EPI) sequence with the following parameters: TR = 4550 ms, TE = 80 ms, FOV = 200 × 200 mm^2^, matrix resolution = 110 × 110, slice thickness = 5 mm, number of slices = 20, one scan at *b* = 0 s/mm^2^ and three at *b* = 500 s/mm^2^ in orthogonal directions (diffusion gradient strength, *G*_diff_ = 110 mT/m gradient time, *δ* = 10 ms, gradient separation, Δ = 20 ms), four averages, total measurement time (including a dummy scan for magnetization preparation) 2 min 30 s. Additionally, a generalized auto-calibrating partially parallel acquisition (GRAPPA) parallel imaging acceleration factor of 2 in the right-left direction and elliptical sampling were used to reduce acquisition time.

### Data processing

#### Semi-quantitative analysis including the whole-organ MRI score and cartilage volume

**Fig. 2:**
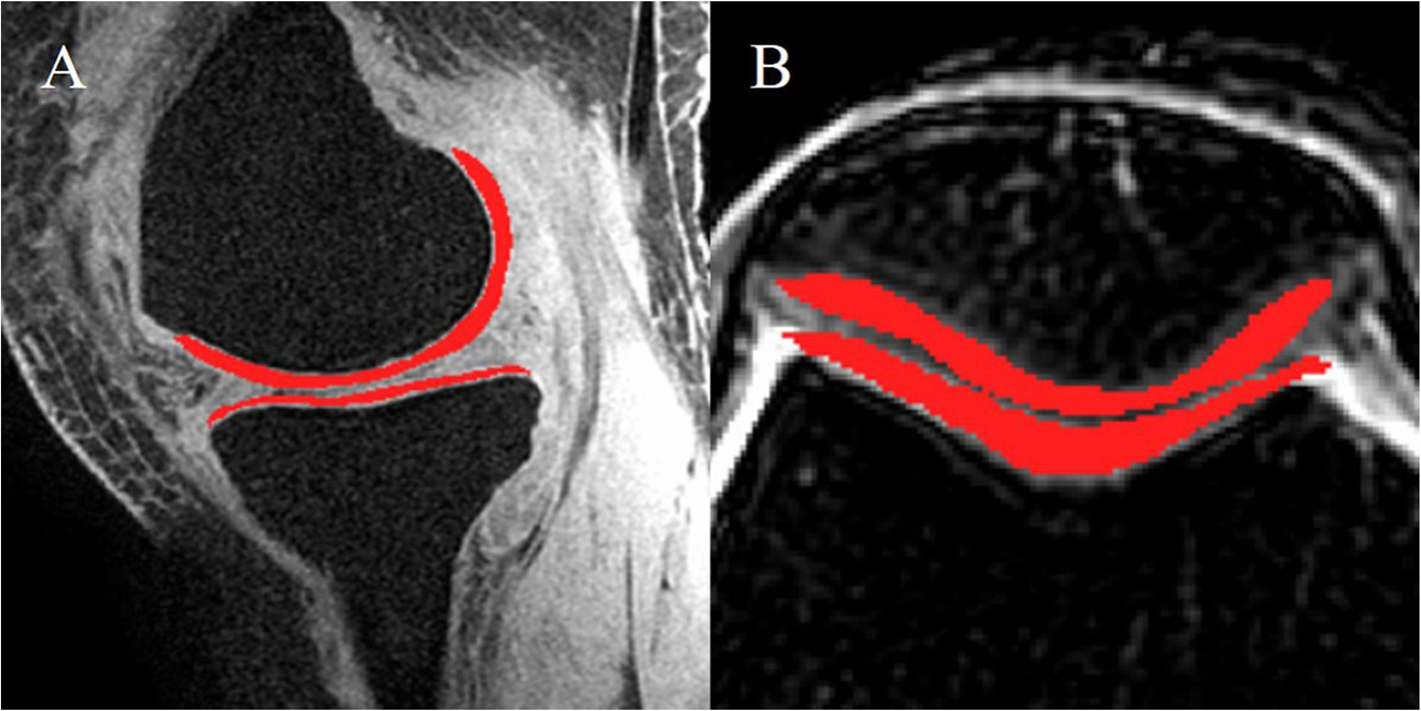
The manually traced ROIs contain the cartilage on the 3D fat-suppressed SPGR for measuring cartilage volume (**a**) and transverse fat-suppressed T2-weighted images for quantitative MRI measures (**b**)

Two radiologists used the whole-organ MRI score systems (WORMS) to semi-quantify the severity of cartilage damage to the signal on sagittal T1-weighted, two-dimensional proton density–weighted, and fat-saturated fast spin-echo T2-weighted images. Two radiologists unaware of the subject’s history manually drew regions of interest (ROIs) of the whole cartilage on the fat-suppressed 3D SPGR images using ITK-SNAP software (Fig. 2a). The ROIs were drawn on all sections where the cartilage was visible, including medial femoral condyle (MF), lateral condyle (LF), femoral inter-condylar (T), medial tibia (MT), lateral tibia (LT), and patella (P). To minimize a partial volume effect, these sections never included the most inferior or most superior slice on which the cartilage was defined and voxels at the tissue boundaries were excluded. The ROIs were checked by osteologists before statistics.

#### Quantitative analysis including relaxometry measurements and ADC/FA values

Relaxation images were obtained by post processing of workstations (version AW4.6) including transverse T2, T1rho, and T2star (or R2star) images, and subsequent images of DWI (ADC and FA mappings). ROIs were segmented on fat-suppressed T2-weighted images (Fig. 2b) on sections with the thickest cartilage according to methods above. To maintain accuracy in quantitative evaluation, the exact same ROIs drawn on the fat-suppressed T2-weighted images were applied to T2, T1rho, T2star, R2star, ADC, and FA maps. The averages of measurements in the whole ROI were calculated for each subject, and the final values were defined as the mean values obtained by two blinded radiologists.

#### Statistical analysis

Statistical analyses were carried out using SPSS for Windows version 23.0 (IBM SPSS Statistics, Chicago, IL). *T* test was used for normally distributed data, and Wilcoxon signed-rank test for nonnormally. The interobserver variability of segmentation was tested using the intraclass correlation coefficient (ICC) for the two observers. Changes from base time in all measures that were scale variables were determined with a paired *t* test (two-tailed) followed by Mann-Whitney *U* tests. The differences between the three dose groups were compared using one-way analysis of variance (ANOVA), followed by Tukey’s multiple comparison test undertaken when significant differences in means were observed. We used the difference before and after treatment (*D* values) as the object to avoid errors caused by individual differences. Two-tailed Pearson correlation analysis was used to investigate correlations between MRI measures and the WOMAC scores. A *P* value of less than 0.05 was considered statistically significant.

## Results

### Longitudinal change during the trial

**Fig. 3:**
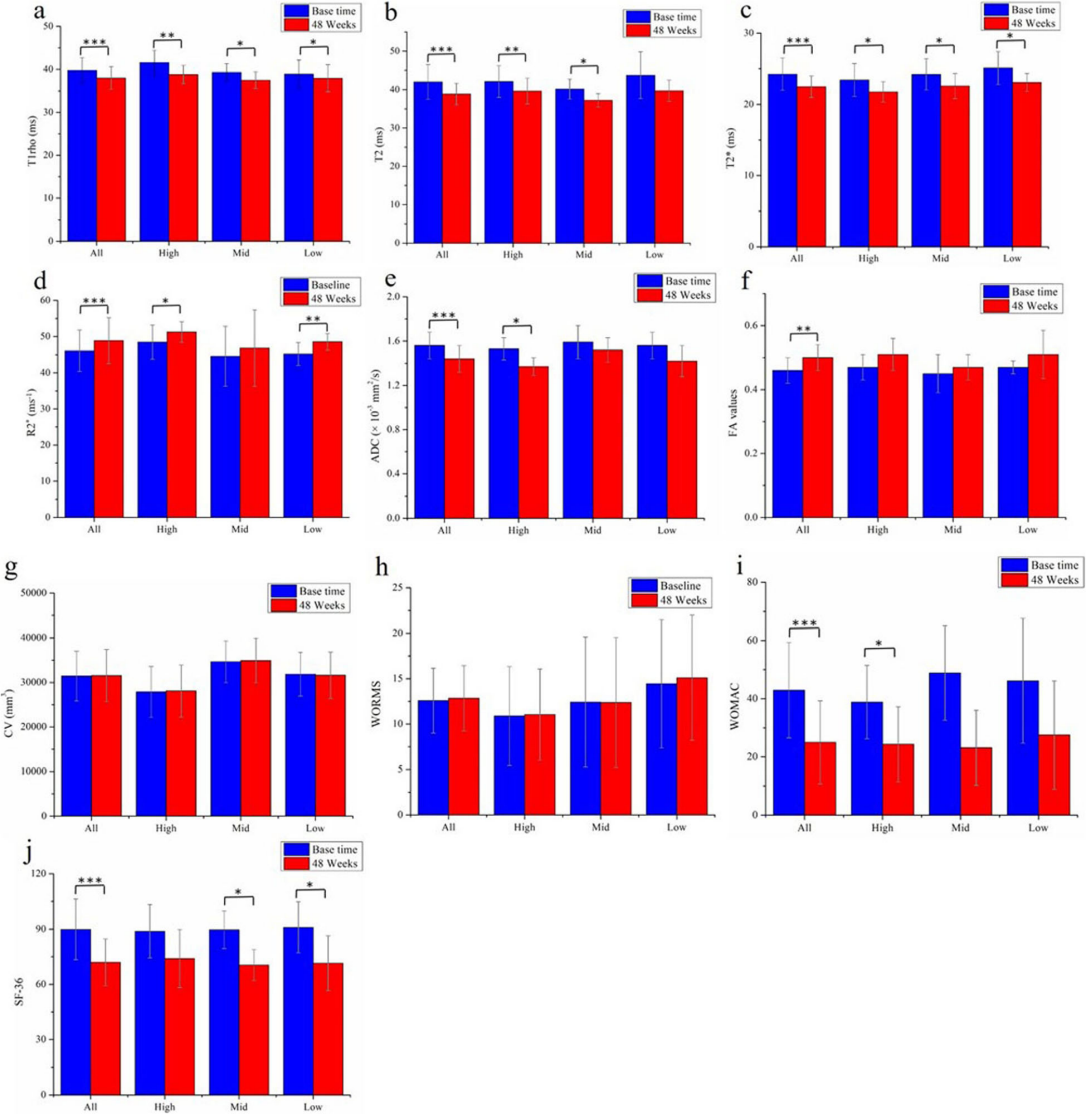
**a**–**j** Comparison of mean measurement values in the articular cartilage of three dose groups

Longitudinal change of all patients between base time and 48 weeks in compositional MRI parameters for the ROIs and clinical scores is summarized in Table 4 and Fig. 3. Compared with base time, there were significant differences in quantitative MRI values after 48 weeks of treatment, as reflected in significant decreases of T1rho (*P* <  0.0001, Fig. 3a), T2 (*P* = 0.0001, Fig. 3b), T2star (*P* <  0.0001, Fig. 3c), and ADC values (*P* = 0.0003, Fig. 3e), whereas increases of R2star (*P* = 0.0001, Fig. 3d) and FA values (*P* = 0.002, Fig. 3f). Significant reduction was also observed in WOMAC (*P* <  0.0001, Fig. 3i) and SF-36 scores (*P* <  0.0001, Fig. 3j), but not in semi-quantitative methods including WORMS (*P* = 0.146, Fig. 3h) and CV (*P* = 0.781, Fig. 3g).

**Table 4:** Longitudinal change between base time and 48 weeks in compositional MRI measurements and clinical outcomes

Measurement	Base time	48 weeks	*P* value
T1rho (ms)	39.73 ± 2.97	37.99 ± 2.61	< 0.0001*
T2 (ms)	42.01 ± 4.50	38.86 ± 2.82	0.0001*
T2star (ms)	24.25 ± 2.25	22.49 ± 1.52	< 0.0001*
R2star (s^−1^)	46.08 ± 5.70	48.87 ± 6.35	0.0001*
ADC (×10^−3^ mm^2^/s)	1.56 ± 0.12	1.44 ± 0.12	0.0003*
FA	0.46 ± 0.04	0.50 ± 0.04	0.002*
WORMS	12.58 ± 3.57	12.84 ± 3.61	0.146
CV (mm^3^)	31,441 ± 5578	31,517 ± 5828	0.781
WOMAC	42.94 ± 16.42	25 ± 14.26	< 0.0001*
SF-36	89.83 ± 12.19	72 ± 12.69	< 0.0001*

Values are given as mean ± standard deviation of the mean

*Significantly different between two groups (*P* < 0.05)

### Sensitivities of measurements to the changes

**Fig. 4:**
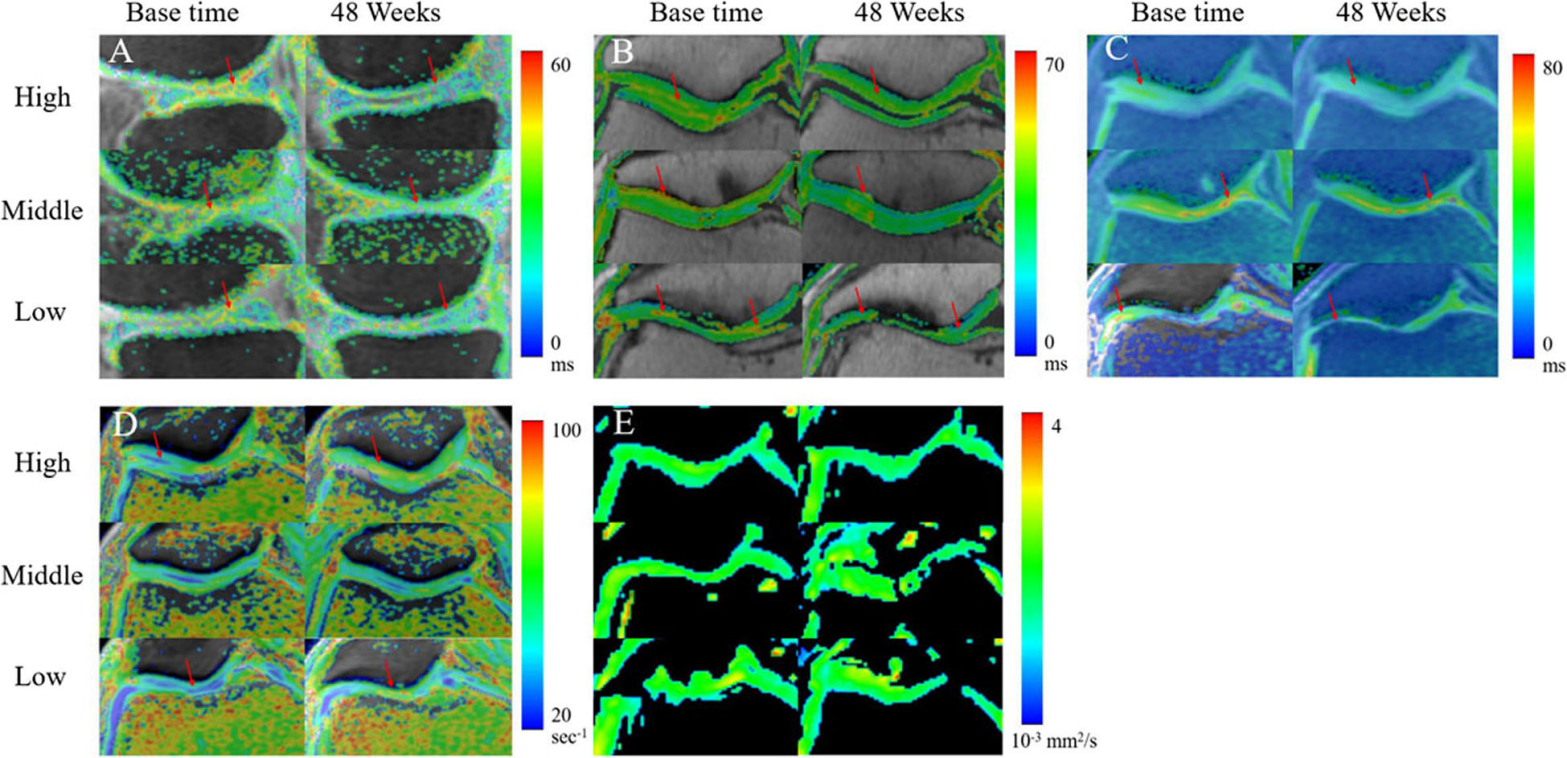
Representative images of quantitative MRI mappings of three patients from high (60 years, F), middle (68 years, M), and low (65 years, M) dose groups. For the same patient, the change (red arrow) of the T1rho (**a**) image was more obvious than that of the T2 (**b**), T2star (**c**), R2 star (**d**), and ADC (**e**) images. Changes of T1rho values between two examination time points in the patient from the high-dose group were more pronounced than those from the other two groups, whereas this phenomenon was not found in other MRI images

Representative images of quantitative MRI mappings of three patients from high (60 years, F), middle (68 years, M), and low (65 years, M) dose groups were shown in Fig. 4. The differences of dose groups on cartilage quality improvement with compositional MR measurements and clinical outcomes are summarized in Table 5 and Fig. 3. For the same patient, the change (red arrow) of the T1rho image was more obvious than that of the T2, T2star, R2 star, and ADC images (Fig. 4). These techniques showed different sensitivity to the changes of three dose groups, of which T1rho and T2star differentiated improvement of cartilage in all three dose groups (*P* = 0.002, 0.002, 0.038 for T1rho values of high, middle, low groups respectively, and *P* = 0.011, 0.012, 0.012 for T2star values).T2, R2star, and SF-36 detected significant changes in two groups. Significant reductions were found in T2 values of the high (*P* = 0.003) and middle (*P* = 0.003) dose groups, whereas not in the low group. The difference of R2star values remained significant in both high (*P* = 0.021) and low (*P* = 0.005) dose groups, not in the middle group. SF-36 also detected significant changes in two groups, middle (*P* = 0.005) and low (*P* = 0.04) dose groups, not in the high group. ADC and WOMAC detected significant changes only in one group (*P* = 0.026 for ADC value in the high group, *P* = 0.035 for WOMAC score in the middle group). However, no significant results were found in FA value, WORMS score, and CV of all dose groups.

**Table 5:** Longitudinal change of comprehensive MRI measurements and clinical outcomes for three dose groups during the trial

Measurement	Group	Base time	48 weeks	*P* value
T1rho (ms)	High	41.55 ± 2.86	38.82 ± 2.11	0.002*
	Mid	39.30 ± 2.01	37.48 ± 1.93	0.002*
	Low	38.91 ± 3.24	37.94 ± 3.13	0.038*
T2 (ms)	High	42.12 ± 4.13	39.65 ± 3.37	0.003*
	Mid	40.16 ± 2.60	37.21 ± 1.73	0.003*
	Low	43.77 ± 6.09	39.73 ± 2.78	0.075
T2star (ms)	High	23.43 ± 2.30	21.77 ± 1.41	0.011*
	Mid	24.22 ± 2.18	22.60 ± 1.77	0.012*
	Low	25.11 ± 2.32	23.09 ± 1.27	0.012*
R2star (s^−1^)	High	48.47 ± 4.69	51.25 ± 2.85	0.021*
	Mid	44.57 ± 8.26	46.80 ± 10.57	0.180
	Low	45.21 ± 3.16	48.57 ± 2.29	0.005*
ADC (×10^−3^ mm^2^/s)	High	1.53 ± 0.10	1.37 ± 0.08	0.026*
	Mid	1.59 ± 0.15	1.52 ± 0.11	0.112
	Low	1.56 ± 0.12	1.42 ± 0.14	0.052
FA	High	0.47 ± 0.04	0.51 ± 0.05	0.05
	Mid	0.45 ± 0.06	0.47 ± 0.04	0.225
	Low	0.47 ± 0.02	0.51 ± 0.03	0.075
WORMS	High	10.88 ± 5.45	11.04 ± 5.02	0.918
	Mid	12.42 ± 7.16	12.38 ± 7.17	0.985
	Low	14.43 ± 7.06	15.10 ± 6.90	0.758
CV	High	27,863.12 ± 5688.74	28,073.37 ± 5871.8	0.599
	Mid	34,645.82 ± 4666.97	34,870.24 ± 4991.32	0.758
	Low	31,815.91 ± 4879.96	31,607.52 ± 5238.41	0.514
WOMAC	High	38.83 ± 12.61	24.33 ± 12.88	0.077
	Mid	48.83 ± 16.22	23.17 ± 12.89	0.035*
	Low	46.17 ± 21.48	27.50 ± 18.64	0.139
SF-36	High	88.83 ± 14.41	74.0 ± 15.74	0.119
	Mid	89.67 ± 10.21	70.5 ± 8.41	0.005*
	Low	91.0 ± 13.80	71.5 ± 14.88	0.040*

Values are given as mean ± standard deviation of the mean

*Significantly different between two groups (*P* < 0.05)

### The results of *D* value analysis and multiple comparison

The *D* value analysis of three dose groups before and after treatment showed that there were significant differences in T1rho values among three dose groups (*F* = 6.31, *P* = 0.025, Table 6), but no significant differences in other measurements (Table 6). This was consistent with results shown in Fig. 3 that the changes of T1rho values between two examination time points in the patient from the high-dose group were more pronounced than those from other two groups, whereas this phenomenon was not found in other MRI images. And as the results of multiple comparisons shown, no significant differences were found among the three groups in measurements except for T1rho. The *D* value of T1rho in the high-dose group was significantly higher than that in the low-dose group (2.73 ± 0.40 ms vs 0.49 ± 0.49 ms, *P* = 0.004, Table 6). In other words, the reduction of T1 rho value in the high-dose group was significantly more than that in the low-dose group, whereas no significant differences were found between high- and middle-dose groups (2.73 ± 0.40 ms vs 1.82 ± 0.40, *P* = 0.129) or middle- and low-dose groups (1.82 ± 0.40 vs 0.49 ± 0.49 ms, *P* = 0.056). Also, there were no significant differences in other *D* values of measurement between three groups.

**Table 6:** The results of *D* value analysis and multiple comparison of three dose groups

Measurement	High	Mid	Low	*F* value	*P* value
T1rho (ms)	2.73 ± 0.40	1.82 ± 0.40	0.49 ± 0.49	6.31	0.025*
	Multiple comparison		High vs low		0.004*
			High vs mid		0.129
			Mid vs low		0.056
T2 (ms)	2.47 ± 1.12	2.95 ± 1.12	4.04 ± 1.11	0.517	0.606
	Multiple comparison		High vs low		0.337
			High vs mid		0.764
			Mid vs low		0.502
T2star (ms)	1.66 ± 0.46	1.62 ± 0.46	2.01 ± 0.46	0.222	0.804
	Multiple comparison		High vs low		0.565
			High vs mid		0.951
			Mid vs low		0.554
R2star (s^−1^)	− 2.78 ± 1.04	− 2.23 ± 1.04	− 3.36 ± 1.04	0.298	0.747
	Multiple comparison		High vs low		0.699
			High vs mid		0.710
			Mid vs low		0.452
ADC (×10^−3^ mm^2^/s)	0.16 ± 0.047	0.07 ± 0.047	0.13 ± 0.047	0.956	0.407
	Multiple comparison		High vs low		0.664
			High vs mid		0.195
			Mid vs low		0.376
FA	− 0.045 ± 0.016	− 0.02 ± 0.01	0.037 ± 0.016	0.627	0.548
	Multiple comparison		High vs low		0.737
			High vs mid		0.291
			Mid vs low		0.463
WORMS	− 0.167 ± 0.75	0.042 ± 0.77	− 0.67 ± 0.74	1.571	0.240
	Multiple comparison		High vs low		0.394
			High vs mid		0.438
			Mid vs low		0.398
CV	− 210.25 ± 485.2	− 224.42 ± 485	− 208.38 ± 485.23	0.257	0.777
	Multiple comparison		High vs low		0.551
			High vs mid		0.984
			Mid vs low		0.538
WOMAC	14.5 ± 5.5	20.67 ± 5.54	18.67 ± 5.54	0.322	0.729
	Multiple comparison		High vs low		0.857
			High vs mid		0.717
			Mid vs low		0.965
SF-36	14.83 ± 14.13	19.17 ± 13.79	19.50 ± 10.97	0.239	0.790
	Multiple comparison		High vs low		0.812
			High vs mid		0.835
			Mid vs low		0.999

Values are given as mean ± standard deviation of the mean

*Significantly different between two groups (*P* < 0.05)

### Clinical outcomes

**Fig. 5:**
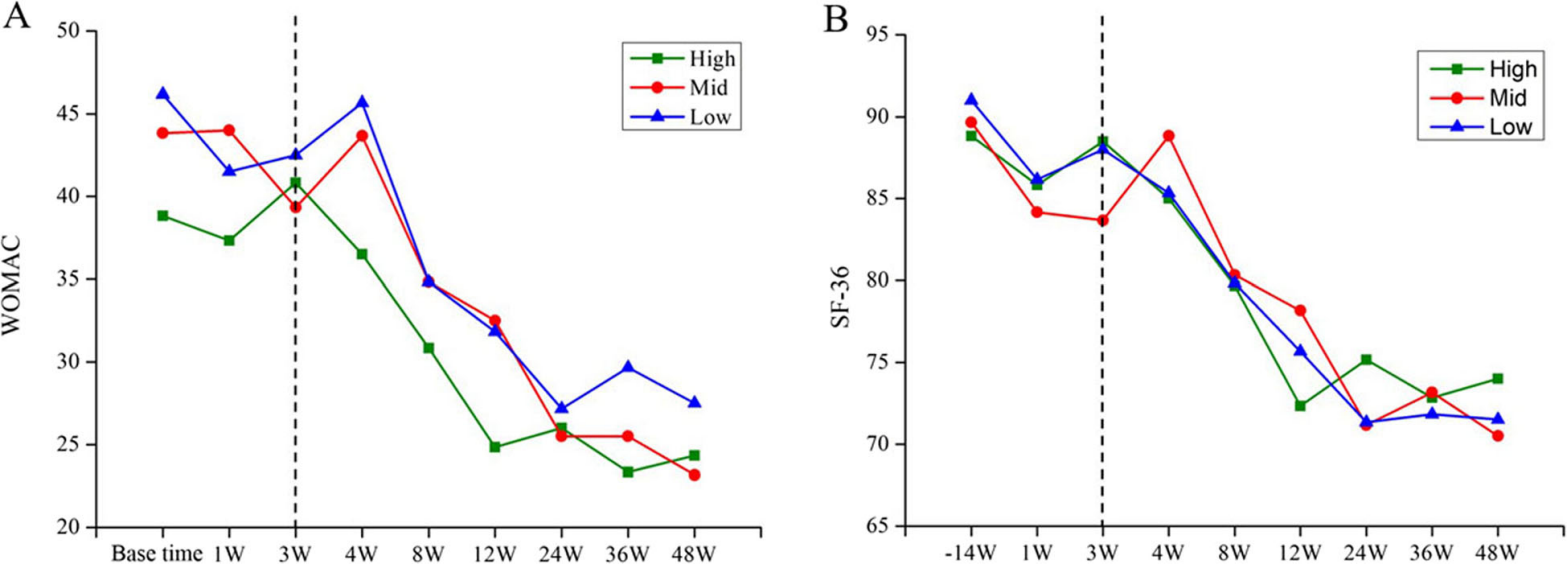
WOMAC pain (**a**) and SF-16 life quality (**b**) improvement during the study

Compared with base time, patients in all dose groups demonstrated significantly clinical pain reduction (approximately 40%) in WOMAC score (42.94 ± 16.42 vs 25 ± 14.26, *P* = 0.001, Table 4, Fig. 3) and improvement (approximately 20%) of quality of life in SF-36 score (89.83 ± 12.19 vs 72 ± 12.69, *P* <  0.001, Table 4, Fig. 3i, j), which were coincident with the longitudinal changes of clinical evaluation from base time to termination (Fig. 5). As shown in Fig. 5a and b, there were a clinical improvement in all dose groups with the treatment of allogeneic haMPCs, particularly after third weeks (second injection), and a tendency to be flat after 24 weeks. Notably, the trends in clinical scoring of the three groups were similar to each other, and the result of multiple comparisons further verified no significant differences were between the three groups of WOMAC (*F* = 0.322, *P* = 0.729, Table 6) between three groups, nor as SF-36 (*F* = 0.239, *P* = 0.790, Table 6).

## Discussion

We used multimodal MRI techniques, combined with clinical outcomes, to evaluate the potential of repair cartilage with allogeneic haMPCs in patients with KOA. In our study, significant differences were observed in quantitative measurements in patients of three dose groups, including T1rho, T2, T2star, R2star, and ADC values, suggesting a possible compositional changes of the cartilage with this innovative treatment (Figs. 3 and 4, Tables 4 and 5). Significant reduction in WOMAC and SF-36 scores showed that the symptoms might be alleviated to some extent with the treatment of allogeneic haMPCs (Figs. 3 and 5). In addition, we also focused on the sensibilities of multi-parametric mappings to detect compositional or structural changes of the cartilage and found that T1rho mapping was most sensitive to differentiate difference between three dose groups.

As we known, the articular cartilage consists of approximately 70–80% fluid and 20–30% solid extracellular matrix (ECM) [34]. The ECM is mainly composed of proteoglycan (PG), glycosaminoglycan (GAG), and type II collagen [3, 35, 36], which provides a motion-restricted environment for water molecules [29]. Histological and biochemical alterations of cartilage ECM involve disruption of the collagen network, decrease in PG or GAG content, and increase in permeability to water [37]. Studies have showed that MSC/MPCs were able to restore the degeneration of ECM by the ability of regeneration and differentiation, which contributed to the repair of the damaged articular cartilage through homing, engraftment, production of cartilage matrix, and reduction of local inflammation [21, 38–40]. Wen et al. have demonstrated that haMPCs could prevent the secretion of type II collagen degraded enzyme, called MMP-13, and in turn rescue type II collagen degradation [15].

MRI studies have reported the decrease of the content of ECM components will lead to an increase of T1rho [41, 42], and moreover, Tsushima et al. have demonstrated the linear relationship between them [43]. In vitro T2 relaxation studies have shown increased cartilage T2 is associated with an increase in water content [44] and a decrease in collagen content [45], and also shown a close relationship between T2 and the architecture of collagen [46]. In our study, a significant reduction in T1rho of three dose groups (Table 5, Figs. 3a and 4a) might indicate an increase of the content of ECM components and relief of cartilage degeneration. Also, significantly lower T2 values were found in cartilage repair tissue at 48 weeks which might indicate the decrease in water content or increase in collagen content in the ECM. Notably, the result of the *D* value analysis showed T1rho imaging exhibits the strongest sensitivity to detect ultra-structural tissue alterations between high-, middle-, and low-dose groups (*F* = 6.31, *P* = 0.025, Table 6). This result was consistent with recent studies that suggested T1rho mapping seems to be more sensitive in detecting early stages of cartilage degeneration than quantitative T2 [47, 48]. In principle, T1rho constant relaxation time represents the transverse magnetization decay in B1 field strength produced by a spin-lock radiofrequency (RF) pulse [37, 49], which reflects the low-frequency (in the kHz range) interactions between motion-restricted water molecules and local macromolecular environment, such as PG, GAG, and collagen II [37]. Therefore, as Holtzman and Teologis concluded that T1rho might reflect differential maturation of the repair tissue for its GAG and collagen specificity [50, 51].

In comparison with T2 mapping, T2star (the reciprocal of R2 star) mapping is acquired with gradient echo sequence, more time efficient than spin-echo sequence. T2star values are associated with the local field inhomogeneities and susceptibility, caused by changes of restricted water mobility in the ECM [28]. We found a significant decrease in T2star values of all dose groups at 48 weeks compared with the base time (Fig. 4b), which was consistent with one study on arthrosis of the ankle [52]. However, one study showed the opposite result that decrease in T2star values was shown to correlate with morphological cartilage damage [53]. No significant difference of *D* value analysis of T2star was found between the three groups (Table 6), suggesting T2star mapping was less sensitive than T1 rho mapping in differentiating difference between three dose groups. Therefore, more scientific evidence is needed to definitively determine the meaning and clinical significance of T2star values and their correlation with cartilage degeneration.

Diffusion imaging may supplement other quantitative techniques for evaluating cartilage degeneration and monitoring its repair following surgery [54–56]. Recent studies reported that ADC measurement correlates with the proteoglycan content in the cartilage. In our results, significant reduction of ADC in the high-dose group at 48 weeks (Fig. 3e) indicated a decrease of diffusivity of water molecules within the cartilage ECM, which might associate with the relief of structural degradation of the ECM. However, no significant changes were found in other two groups. That was consistent with our result of T1rho in *D* value analysis. Thus, it may be known, the effects of three doses of drugs on cartilage repair were different.

In the current study, quantitative methods were more sensitive than semi-quantitative (included WORMS and CV) to detect longitudinal change during the trial (Table 4) and to differentiate improvement of cartilage in high-, middle-, and low-dose groups (Table 5). This result was consisted with a head-to-head comparison of semi-quantitative and quantitative approaches for the assessment of cartilage damage [57]. The results of semi-quantitative assessment showed its potential weakness of insensitive to ultra-structural tissue alterations in articular cartilage, which were consistent with previous conclusions [23, 24, 57]. Nevertheless, it was uncertain whether the comparison results of WORMS and CV values were affected by individual differences, especially in the case of unnegligible standard deviation in our study. Our results showed clinical improvement in all dose groups with the injection of allogeneic haMPCs (Fig. 3a, b), demonstrating the efficacy of the treatment. Despite the reduction in WOMAC and SF-36 scores in the entire trial period, no statistically significant difference in clinical pain scores was observed between each group at any time point in this study (Fig. 3i, j), which was consistent with the previous study [58]. Additionally, no correlation was identified between compositional MR measurements and clinical changes including the volume of cartilage, WOMAC, and SF-36 scores. This was consistent with previous study [7, 59], whereas someone showed the opposite results [60]. Therefore, more scientific evidence is needed to determine the reason of this discrepancy.

The results of the *D* value analysis of T1rho values demonstrated that dose is one of crucial factors for the efficacy of the cellular therapy. In addition to the dose, there are many other potential variables affecting the effectiveness of cellular therapies based on MSCs, such as in vitro preparation, including immunogenicity, cryopreservation, donor variance, ex vivo expansion, and senescence [61, 62]. Much effort has been expended in optimizing cell functionality in our paper. To avoid issues related to bovine serum protein, such as prion-related encephalopathy or other xenogeneic intrinsic-immunogenicity infections, we used a potent serum-free medium for culture and expansion of the stem cells. In our study, stem cells were cryopreserved in liquid nitrogen and then thawed cells before transplantation, same as conventional approach in human clinical trials examining the use of MSCs [63]. However, some reports have demonstrated human MSCs display a “heat shock” response to thawing, which has a marked and transient dampening of their immune suppressive properties [61, 64]. For this reason, it is critical to prevent cell immune functionality from being damaged by the “heat shock” and needs to be explored in depth. In addition, immunoregulatory properties of human MSCs may have a significant inter-donor variability which will interfere clinical outcomes. High interferon-gamma responder donor might express desirable interferon-gamma-induced IDO upregulation [65]. To avoid less potent products in subjects participating in pivotal clinical trials, volunteer donors should be screened for immune plasticity in our further investigations [61]. Recent studies have suggested human MSC product performance was associated with the scale of product ex vivo expansion. A limited number of early passage MSCs, such as passage 4 used in this paper, have shown more effective than comparable late passage [61, 66]. Expansion pressure has demonstrated to lead to replicative senescence, and moreover, the senescent MSCs would lose some mesenchymal plasticity and might affect their therapeutic potential [67]. In this scenario, it is necessary to evaluate the cellular senescence of haMPCs before transplantation. Capasso et al. have analyzed the senescence of MSCs comprehensively in aspects of cellular metabolism, autophagy, and proteasome activity [62]. An in-depth evaluation of senescence of haMPCs by Capasso’s method should be performed in our further investigations.

Our study had several limitations. Firstly, the number of subjects was relatively small, so there is a need to encourage large randomized clinical trials. Secondly, there was no zonal evaluation of the articular cartilage. We will use zonal analysis for in-depth assessment of cartilage repair, particularly considering the depth-wise distribution of T1rho, T2, and T2star values. Thirdly, while regeneration of the articular cartilage was clearly identified with MRI measures and the tendency of curves of clinical scores was to be flat after 24 weeks, the 48 weeks of follow-up would be short especially for the assessment of clinical outcomes. Further investigations for longer period would be necessary next. Fourthly, no histological analysis was supplemented. Despite quantitative MR measures and clinical outcomes consistently demonstrated cartilage improvement and pain relief, the healing effects should be confirmed in future studies with histological data. Finally, several details of cell preparation should be improved, such as selection of volunteer donors for immune plasticity and a comprehensive evaluation of senescence of haMPCs.

## Conclusion

In summary, multi-compositional MRI sequences could evaluate the promotion of the repair of cartilage with allogeneic haMPCs in patients with KOA by providing supplementary information of compositional alterations of the articular cartilage, which might be an effective tool for demonstrating efficacy of this new drug and guiding clinical decision-making in the follow-up treatment.

## Data Availability

The datasets used and/or analyzed during the current study are available from the corresponding author upon request.

## Abbreviations

MRI: Magnetic resonance imaging
KOA: Knee osteoarthritis
MSC/MPC: Mesenchymal stem/progenitor cells
haMPCs: Human adipose-derived MPCs
AD-MPCs: Adipose-derived MPCs
BM-MSCs: Bone marrow-derived MPCs
AD-MSCs: Adipose tissue-derived MSCs
GAG: Glycosaminoglycan
ECM: Extracellular matrix
PG: Proteoglycan
DWI: Diffusion weighted imaging
ADC: Apparent diffusion coefficient
WORMS: Whole-organ MRI score
WOMAC: Western Ontario and McMaster Universities Osteoarthritis Index
SF-36: Short-Form-36 questionnaire
3D SPGR: Three-dimensional spoiled gradient recalled echo
ss-EPI: Single-shot spin-echo echo planar imaging
GRAPPA: Generalized auto-calibrating partially parallel acquisition
MF: Medial femoral condyle
LF: Lateral condyle
T: Femoral inter-condylar
MT: Medial tibia
LT: Lateral tibia
P: Patella
ICC: Intraclass correlation coefficient
ANOVA: Analysis of variance
CV: Cartilage volume
PBS: Phosphate-buffered saline
DMEM: Dulbecco’s modified Eagle’s medium
STR: Short tandem repeat
FACS: Fluorescence-activated cell sorting
Ch.P.2015: The 2015 version of the Chinese Pharmacopoeia
